# *Flaviviridae* Viruses and Oxidative Stress: Implications for Viral Pathogenesis

**DOI:** 10.1155/2019/1409582

**Published:** 2019-08-19

**Authors:** Zhenzhen Zhang, Liang Rong, Yi-Ping Li

**Affiliations:** ^1^Institute of Human Virology and Zhongshan School of Medicine, Sun Yat-sen University, Guangzhou 510080, China; ^2^Key Laboratory of Tropical Disease Control (Sun Yat-sen University), Ministry of Education, Guangzhou 510080, China; ^3^Program of Pathobiology, The Fifth Affiliated Hospital and Zhongshan School of Medicine, Sun Yat-sen University, Guangdong, China

## Abstract

Oxidative stress is induced once the balance of generation and neutralization of reactive oxygen species (ROS) is broken in the cell, and it plays crucial roles in a variety of natural and diseased processes. Infections of *Flaviviridae* viruses trigger oxidative stress, which affects both the cellular metabolism and the life cycle of the viruses. Oxidative stress associated with specific viral proteins, experimental culture systems, and patient infections, as well as its correlations with the viral pathogenesis attracts much research attention. In this review, we primarily focus on hepatitis C virus (HCV), dengue virus (DENV), Zika virus (ZIKV), Japanese encephalitis virus (JEV), West Nile virus (WNV), and tick-borne encephalitis virus (TBEV) as representatives of *Flaviviridae* viruses and we summarize the mechanisms involved in the relevance of oxidative stress for virus-associated pathogenesis. We discuss the current understanding of the pathogenic mechanisms of oxidative stress induced by *Flaviviridae* viruses and highlight the relevance of autophagy and DNA damage in the life cycle of viruses. Understanding the crosstalk between viral infection and oxidative stress-induced molecular events may offer new avenues for antiviral therapeutics.

## 1. Introduction

The *Flaviviridae* is a family of viruses that comprise more than 100 species. These viruses include those that have been grouped into one of four genera (*Flavivirus*, *Pestivirus*, *Pegivirus*, and *Hepacivirus*) [1], as well as a number of unclassified species. Natural hosts of *Flaviviridae* viruses are primarily humans and other mammals, and the viruses spread mainly through arthropod vectors (e.g., mosquitoes and ticks; *Flavivirus*) and blood-associated transmission (*Hepacivirus*). Medically important *Flaviviridae* viruses include dengue virus (DENV), Zika virus (ZIKV), yellow fever virus (YFV), Japanese encephalitis virus (JEV), West Nile virus (WNV), tick-borne encephalitis virus (TBEV), and hepatitis C virus (HCV) [1]. Major clinical manifestations associated with the infections of these viruses include hemorrhagic syndromes [2], neurological complications (e.g., encephalitis, Guillain-Barré-syndrome (GBS), and microcephaly) [3, 4], and hepatitis [5]. After entry into the cell through receptor-mediated endocytosis, the life cycle of *Flaviviridae* viruses is completed entirely in the cytoplasm. Many host cellular factors have been identified as being involved in the viral infection, and the various steps of the life cycle or viral products interfere with the homeostasis of cellular metabolism, thus triggering a stress pressure to the host cell. In general, cellular metabolism steadily produces reactive oxygen species (ROS), as a byproduct of the normal aerobic metabolism, by a variety of enzymes in mitochondria, endoplasmic reticulum (ER), and peroxisome compartments, and simultaneously the oxide is removed to keep the balance [6, 7]. ROS are reactive chemical species containing oxygen, for example, hydrogen peroxide (H_2_O_2_), superoxide anion (O_2_^•−^), hydroxyl radical (^•^OH), and singlet oxygen (^1^O_2_). In a biological context, ROS are oxidants and mediators of cell injury, disease, homeostasis, and signaling activation [6, 7]. Once the balance between the accumulation of ROS and the removal of the oxide is broken, an oxidative stress is established. The ultimate consequences of oxidative stress are tissue damage, inflammation response, and cell death [8, 9]. To maintain a favorable environment for cell survival and to restore the balance of oxidation and antioxidant systems, the cell generally restricts the use of nutrients and energy through, for example, reducing protein synthesis [10, 11]. It is noted that oxidative stress as a mere imbalance between ROS production and neutralization is being substituted by a concept of redox pathophysiology, which focuses on searching for exact reactions between ROS or oxidation products with exact groups of proteins and the consequences of these reactions.

Thus, an updated definition of “an imbalance between oxidants and antioxidants in favor of the oxidants, leading to a disruption of redox signaling and control and/or molecular damage” is being introduced for oxidative stress [12]. A variety of viral infections have been found to trigger oxidative stress and thus interfere with the normal function of the host cell. Therefore, the maintenance and restoration of a favorable intracellular environment are vitally important for the host to combat against virus infection. Several *Flaviviridae* viruses have been demonstrated to trigger oxidative stress in the infected cell, which include the viruses of the *Flavivirus* genus, DENV [13–15], ZIKV [16, 17], JEV [18–20], WNV [21, 22], and TBEV [23], as well as *Hepacivirus* HCV [24–28].

Among *Flaviviridae* viruses, HCV infection and the induction of oxidative stress have been more extensively studied. HCV is a major cause of liver disease, and majority of infected patients develop chronic infection, which increases the risk of developing liver cirrhosis, liver failure, and eventually hepatocellular carcinoma (HCC). Globally, chronic HCV infection affects 71 million people, and HCV-associated diseases lead to 400,000 deaths per year [29]. Direct-acting antiviral agents (DAAs) have been approved recently for the treatment of HCV infection and have dramatically increased the cure rate up to 90% [30]. However, a vaccine for HCV is still unavailable. Besides, new challenges arising from the limited access and high cost of DAA, emergence of drug resistance, impaired neutralizing immunity, and unawareness of infection, as well as the unceasing progression of HCC and risk of reinfection after virus clearance have compromised the global eradication and elimination of HCV infection [31]. Many reports have described the production of ROS in HCV-infected cells and in the liver tissue and lymphocytes from HCV-infected patients [24, 27, 28, 32, 33]. Almost all HCV proteins have been demonstrated to be involved in the induction of oxidative stress, of which core and NS5A proteins are evidenced as the main inducers.

DENV, ZIKV, JEV, WNV, and YFV are mosquito-transmitted viruses, mainly prevalent in tropical and subtropical regions, and cause major health and economic problems. Although most of the infections are asymptomatic or present as mild illness, a portion of the infected patients develops severe symptoms and may result in death. DENV infection could lead to severe symptoms, including dengue hemorrhagic fever (DHF) and dengue shock syndrome (DSS) [34–36]. ZIKV infection may cause serious neurological complications, including GBS in adults and the microcephaly birth defect in infants [37–39]. The birth defect along with its sexual and transplacental transmission makes ZIKV unique and distinct from other flaviviruses [40]. JEV infection causes acute encephalitis and, frequently, neurological sequelae, which lead to the loss of more disability-adjusted life years (DALY) than any other arthropod-borne virus [41, 42]. A small portion of WNV and TBEV (tick transmitted) can also result in severe neurological symptoms (e.g. encephalitis, meningitis, or meningoencephalitis) and may lead to mortality [42, 43]. To date, there is no specific antiviral treatment available for the infections of these flaviviruses. Vaccines for human use are only available for JEV and DENV. However, JEV vaccines are not effective against all clinical JEV isolates, while DENV vaccines can only be used in dengue endemic regions and in the individuals of a specific age range [44].

Accumulating evidences have linked the oxidative stress to the pathogenesis of these vector-borne flaviviruses [45]. In this review, we focus on the infection of six viruses, including HCV, DENV, ZIKV, JEV, WNV, and TBEV. We aim to summarize the reported data regarding the role of oxidative stress in viral pathogenesis and the crosstalk between virus, elevated ROS levels, and oxidative stress-induced molecular events. In addition, autophagy and DNA damage are representatively summarized for HCV, DENV, and ZIKV.

## 2. Virion Structure and Life Cycle of *Flaviviridae* Viruses

Although *Flaviviridae* viruses share a high similarity in the replication cycle, some specific features differ from each other in terms of virion, genome, replication, etc. The virion of *Flaviviridae* viruses has enveloped, icosahedral, and spherical geometries with a diameter of about 40-60 nm [1, 46]. The viral genome is a positive single-stranded RNA of 9.6-12.3 kb, consisting of one large open reading frame (ORF) flanked by untranslated regions (UTRs) at each end. In the 5′UTR, the *Flavivirus* genus carries a methylated nucleotide cap (type I cap), while viruses from other genera possess an internal ribosome entry site (IRES), important for the initiation of RNA translation [1, 47]. All members of the family lack a 3′-terminal poly(A) tract. After entry into the cell, the genomic RNA is uncoated and is directly recognized as a mRNA template by the translation machinery of the host cell to synthesize a large polyprotein precursor. The polyprotein is co- or posttranslationally processed by viral and cellular proteases to produce individual viral proteins, including structural and nonstructural (NS) proteins. Structural proteins form the virion, which is usually comprised of a single core protein (capsid in flaviviruses; C) and two or three envelope proteins. NS proteins are mainly involved in building up the replication complex and regulating RNA replication, viral assembly, and host responses. NS1 is unique to flaviviruses and is the only NS protein residing in the lumen of the ER. The NS1 and NS2A of flaviviruses are required for RNA replication and production of an infectious particle. The NS2B of flaviviruses or the NS4A of HCV acts as a cofactor for the NS3 protease and recruits NS3 to the ER membrane. NS3 is a multifunctional protein with serine protease, RNA helicase, and nucleoside triphosphatase (NTPase) activities that are essential for RNA replication. The NS4A of flaviviruses is an integral membrane protein involved in membrane rearrangement to form the viral replication complex, while the NS4B of HCV functions in this aspect. The NS5 of flaviviruses and the NS5B of HCV both possess RNA-dependent RNA polymerase activity responsible for viral RNA synthesis. In addition, HCV NS5A is a phosphoprotein involved in the interactions with host factors and in RNA binding. HCV p7 is a small protein with ion channel activity. p7 and NS2 of HCV support the assembly and release of virions, which may diverge mechanically from the flaviviruses [1]. Despite a high degree of similarity in the genome organization and the mechanism of RNA replication, important differences exist between flaviviruses and hepacivirus, including the way in which these viruses utilize cellular resources to favor their propagation and possible clinical outcome, likely reflecting the use of distinct host cellular pathways.

## 3. Source and Cellular Response of ROS

ROS are byproducts of aerobic metabolism in various cell compartments, including the mitochondria and ER, and function as fine-tuning modulators of numerous normal physiological processes [7]. It is well known that ROS could be both beneficial and deleterious to the cell, depending on the source and cellular response of ROS [48]. Oxidative stress induced by unbalanced ROS production and scavenging contributes to physiological disorders, such as metabolic dysfunction and neurodegeneration [49], inflammatory activation [50], and infectious diseases [8, 51]. As a natural defense system, the cell regularly produces antioxidants or adopt other mechanisms to eliminate the ROS to keep the balance of antioxidation and oxidation. Thus, the development of antioxidants and strategies are emerging as potential therapeutic approaches for diseases related to the oxidative stress.

### 3.1. Mitochondria: Source and Target of ROS

Mitochondria are the powerhouse of eukaryotic cells responsible for the majority of oxygen consumption inside the cell, supplying 90% of cellular energy; they are also the major producers of intracellular ROS. Mitochondrial respiration depends on electron transfer and a proton gradient to drive ATP synthesis, and in this process, ROS are produced as natural byproducts. The superoxide is primarily produced by an electron leak from electron transport chains (ETC) in mitochondria and through the oxidation of nicotinamide-adenine dinucleotide phosphate (NADPH) by NAPH oxidases (NOXs), as well as by enzymes including pyruvate and 2-oxoglutarate dehydrogenases [52–56]. The superoxide produced is rapidly converted into hydrogen peroxide by superoxide dismutases (SODs) and into hydroxyl radicals through reactions with metal cations within the cell [56]. The ROS produced mostly target the mitochondrial DNA (mtDNA) and are believed to induce mtDNA degradation, owing to the close proximity of the mitochondrial genome to the ETC. In addition to mtDNA damage, excessive ROS accumulation in the mitochondria could trigger mitochondrial outer membrane permeabilization (MOMP), which facilitates the release of cytochrome C (apoptosis activator) into the cytosol and the activation of caspase-3 (key apoptosis effector) [57–59]. Upon induction of MOMP, the capacity of mitochondrial Ca^2+^ buffering is decreased. As a consequence, mitochondrial Ca^2+^ is overloaded, and thus ROS is generated. Although it is incompletely certain whether increased ROS are a consequence of mitochondrial dysfunction or damage, it is clear that the increased ROS levels and oxidative stress that resulted from mitochondrial dysfunction have now been convincingly correlated to a variety of human pathological states, including infection of pathogens [60, 61].

### 3.2. Endoplasmic Reticulum (ER) and ROS

ER is an organelle containing complex and dynamic tubules, which serve as the site of the storage of Ca^2+^, folding and secretory pathways of proteins, and lipid synthesis. Growing evidences have shown that the oxidation of proteins in ER is responsible for the generation of ROS that cause oxidative stress [58, 62, 63]. Considering the intimate cross-talks between ER and mitochondria [64], which form very dynamic platforms termed mitochondria-associated membranes (MAM), the ER-associated oxidative stress could induce mitochondrial Ca^2+^ overload, unfolding protein response (UPR), and autophagy, as well as involvements in virus infection [65, 66]. Such stress responses may switch a process from being physiologically beneficial to one that signals cell damage [58].

### 3.3. Antioxidant Defense Systems

To counteract oxidative stress, eukaryotic cells have evolved antioxidant defense systems, which are composed of antioxidant molecules that inhibit oxidants from reacting with other molecules by ETC, thereby minimizing damage and maintaining cellular redox homeostasis [67, 68]. The molecules of antioxidant defense systems can be endogenous and exogenous. The endogenous (or adaptive) antioxidants consist of both enzymatic (e.g., peroxiredoxins, SOD, catalase, and glutathione peroxidase) and nonenzymatic components (e.g. vitamin C, vitamin E, uric acid, metal binding proteins, polyamines, bilirubin, carotenoids, and glutathione). However, only vitamin E is found to act as a ROS scavenger with visible efficacy, whereas other molecules, albeit they can scavenge ROS *in vitro*, do not act so *in vivo*, but are considered to affect redox-sensitive pathways [69]. Yet, it has also been reported that the antioxidant effects of vitamin E are only displayed *in vitro* and not *in vivo*; thus, it remains a matter of controversy as to whether or not vitamin E is useful for protecting the body from ROS-induced oxidative stress [70]. Many of these antioxidants are encoded by the Nrf2- (nuclear factor erythroid 2-related factor 2-) Keap1 (kelch-like ECH-associated protein 1) pathway [67, 71–74]. Nrf2 is a transcription factor that regulates the expression of numerous ROS detoxifying and antioxidant genes [72, 75]. Under basal physiology, Nrf2 is sequestered in the cytosol by interacting with Keap1, which facilitates the ubiquitination of Nrf2 and proteasomal degradation, thus limiting the expression of Nrf2-regulated genes. Upon cellular oxidative stress, the conformation of Keap1 is changed and thus Nrf2 is released and no longer degraded. Newly synthesized Nrf2 is accumulated and translocated to the nucleus, where it forms a heterodimer with small Maf (sMaf) proteins and binds to the antioxidant response element (ARE). The binding of Nrf2 to ARE initiates ARE-dependent transcriptions of antioxidant defense enzymes, thus building up antioxidant defenses to attenuate cellular oxidative stress and to return the cell to a basal state. Exogenous antioxidants are derived from the diet and supplements, such as tiron (4,5-dihydroxy-1,3-benzenedisulfonic acid; a ROS scavenger), carotenoids, and flavonoids. Most of the endogenous and exogenous antioxidants are localized within the cytosol, and a small portion are localized within the mitochondria [76]. Antioxidant systems are manipulated by *Flaviviridae* viruses and are associated with HCV chronic infection [26] and the immune and apoptotic responses against DENV infection via Nrf2-dependent regulation of ROS production [13]. Although it is a hope and an attempt to modulate the antioxidants for therapeutic purpose, a large challenge exists, mainly in delivering antioxidants to diseased tissue or dysfunctional cells without affecting normal tissue.

## 4. Oxidative Stress and Viral Infections

Oxidative stress induced by virus infection was first described in the study of Sendai virus infection of phagocytes in 1979 [77]. In this study, the binding of the Sendai virus to the cell was demonstrated to quickly activate a membrane-bound NADPH-linked dehydrogenase. This enzyme forms a superoxide anion and hydrogen peroxide, which together with ROS, reacts with polyunsaturated fatty acids, carbohydrates, or artificial easily oxidized substrates such as luminol (5-amino-2,3-dihydro-1,4-phthalazinedione), and finally leads to the induction of chemiluminescence. Since then, many viruses have been demonstrated to cause cell damage by generating ROS and changing redox homeostasis [33, 78–80]. The induction of oxidative stress may also activate the antiviral inflammatory signaling pathways [81–84], thus contributing to viral pathogenesis. For example, DENV infection induces NOX-dependent oxidative stress, which activates interferon regulatory factor 3 (IRF-3), IRF-7, and signal transducer and activator of transcription 1 (STAT-1), as well as nuclear factor kappa-light-chain-enhancer of activated B cell- (NF-*κ*B-) mediated antiviral responses, and associates with the severe damage of virally infected cells [13]. DENV can also induce oxidative stress in liver cells, leading to the production of CCL5 and the activation of the transcriptional regulator CCAAT/enhancer-binding protein *β* (C/EBP*β*) [85]. High levels of circulating proinflammatory cytokines such as IL-1*β* or tumor necrosis factor *α* (TNF-*α*) also correlates with severe dengue fever in DENV-infected patients [86]. Oxidative stress caused by other viruses, such as Rift Valley fever virus (RVFV; a mosquito-borne virus), could also activate inflammatory regulators NF-*κ*B and p53 responses, which in turn modulates cytokine expression, apoptosis, and cell death [82]. The respiratory syncytial virus (RSV) also activates NOX-dependent IRF-3 [87], and NOX2-derived ROS are required for the host cell to trigger an efficient retinoic acid-inducible gene 1- (RIG-I-) mediated activation of IRF-3 and downstream antiviral genes through the regulation of mitochondrial antiviral signaling protein (MAVS) [83]. In turn, ROS may facilitate viral replication, depending on the cell type and the virus involved [51]. Flaviviruses and alphaviruses may use oxidative stress produced during infection to help temporally control genome RNA capping and genome replication [21]. DENV infection is able to activate ER stress-regulated autophagy to limit apoptosis of the cell and thus increase the potential of DENV replication [88]. To counteract the oxidation from the high chemical reactivity of ROS, cells have evolved antioxidant mechanisms to maintain redox homeostasis. Although varying in the ability to induce ROS, viruses share a common pathogenic pathway to defend against oxidative stress. Modulation of Nrf2/ARE signaling is a universal strategy for viruses to trigger antioxidant responses [89].

### 4.1. Oxidative Stress in HCV Infection

Mitochondria are the major sources of ROS inside hepatocytes and liver-resident blood cells. Excessive production of ROS is a leading factor that contributes to liver inflammation, fibrogenesis, and hepatic carcinogenesis [50], which are common sequelae of chronic HCV infection. It is well known that HCV triggers oxidative stress and modifies antioxidant systems, leading to chronic hepatitis C and extrahepatic manifestations such as type 2 diabetes mellitus [90–94], cardiovascular diseases [95–98], autoimmune or lymphoproliferative disorders [99–101], glomerulopathies [102], and neurological diseases [103–107]. Biomarkers of oxidative stress could be detected both in chronic HCV patients and in various *in vitro* systems including replicon systems and infectious cell culture systems [26, 33, 108, 109]. The induction of oxidative stress by HCV has been summarized recently in excellent reviews [26–28, 110]. Most of the HCV proteins are involved in the induction of oxidative stress, including the core, E1, E2, NS3, NS4B, and NS5A [111–121]. It is worth noting that the HCV core protein serves as a key regulator causing calcium perturbations and ROS production, and multiple mechanisms are exploited by the HCV core to induce oxidative stress. The core is localized to mitochondria and MAM with ER and induces the efflux of Ca^2+^ from ER to mitochondria, which causes glutathione (GSH) oxidation and consequent complex I glutathionylation, and thus sustaining and amplifying the oxidized mitochondrial environment [114]. The N terminal of the core (36 residues) induces the production of ROS through transforming growth factor *β*1- (TGF-*β*1-) dependent expression of NOX1 and NOX4, while the rest of the core upregulates ROS transcription via cytochrome P450 2E1 and induces oxidoreductin-1*α*, thus triggering Ca^2+^ efflux from ER to mitochondria via the mitochondrial Ca^2+^ uniporter, leading to the generation of superoxide anions [119]. Both the HCV core and NS5A proteins have been shown to induce ER stress, leading to cytoplasmic Ca^2+^ release via the inhibition of the sarcoplasmic/endoplasmic reticulum calcium ATPase (SERCA) and the induction of a passive leak of Ca^2+^, respectively [33, 122]. NS5A-induced Ca^2+^ release triggers ROS production in mitochondria, leading to the activation of NF-*κ*B and STAT-3 [118]. Envelope proteins E1 and/or E2 induce stress indicator CCAAT/enhancer-binding protein-homologous protein (CHOP) [123] and accumulation of ROS and activation of Nrf2 [124]. E2 upregulates collagen-*α*-related production of ROS in the hepatic stellate cells and contributes to fibrosis [116]. HCV NS3 can trigger oxidative stress in human monocytes via the activation of NADPH oxidases [112]. NS4B induces oxidative stress and associates with the activation of the phosphatidylinositol 3 kinase- (PI3K-) Akt pathway [125]. In addition, HCV infection triggers MOMP accompanied by ROS production, leading to DNA damage and STAT-3 activation, and this activation is achieved by the core, E1, and NS3 proteins [126]. As STAT-3 has been identified as an oncogene, its activation may lead to downstream proliferative responses. Furthermore, the HCV replicon also causes oxidative stress to activate STAT-3 signaling which involves p38 MAPK, JNK, JAK2, and Src kinases, which eventually contributes to the stimulation of HCV RNA replication [109].

In spite of these observations, studies also report the inhibitory effect of elevated ROS levels on viral genome replication. ROS, within the range of a biologically relevant concentration, could suppress HCV RNA replication in human hepatoma cells [127]. HCV replication is susceptible to the cellular redox state [128], and some adaptive mutations that permitted in vitro infection of HCV genomes adaptive mutations permitted by *in vitro* virus production are found to confer resistance to peroxidation [128, 129]; this study also indicates that NS4A and NS5B are involved in the viral susceptibility to lipid peroxidation [128]. Another study reports that besides the induction of oxidative stress, HCV also induces ROS scavenger glutathione peroxidase 4 (GPx4) to control lipid peroxidation and to increase virion infectivity [130]. A recent study describes that a marked increase in oxidative stress in liver cell cultures favors viral replication in the acute phase of HCV *in vitro* infection, while HCV replication and apoptosis are observed at low levels during the chronic phase; concomitantly, the restoration of reducing redox condition is recorded [131]. The conflicting results about the relevance of ROS for the HCV life cycle may be due to the concentration of ROS or the stage of HCV infection.

The modulation of oxidative stress for a therapeutic purpose is being exploited for HCV infection. Promising results have been obtained by modifying the antioxidant defense mechanisms, which include antioxidant agents (mainly the glutathione system and thioredoxin), antioxidant enzyme systems (superoxide dismutase, catalase, GSH peroxidase, and heme oxygenase-1 (HO-1)), and natural and synthetic antioxidants (vitamins C and E, N-acetylcysteine, silymarin, etc.) (reviewed in [132, 133]). The inhibition of glycogen synthase kinase 3*β* (GSK3*β*) enhances the Nrf2 antioxidant response, thus conferring protection in HCV-infected cells and hepatitis C patients [134]. Nevertheless, along with its therapeutic potential, there are concerns on antioxidant therapy. Taking vitamins as an example, several studies report that the serum levels of vitamins (*β*-carotene (provitamin A) and vitamins B, C, and D) are significantly reduced in chronic hepatitis C patients [135–137], but the underlying mechanisms are unclear. These vitamins may have been seriously depleted in the patients; therefore, high doses of primary antioxidants (vitamins) should be administered to restore and, thereafter, to maintain serum and tissue concentrations at normal levels. Thus, the use of antioxidants necessitates early restoration of optimal concentrations in the liver and plasma. However, the results of clinical studies about the efficiency of vitamins for chronic hepatitis C are still controversial [138]. It has been reported that vitamin D2 inhibited HCV RNA replication [139], while vitamin E enhanced HCV RNA replication [140]. No significant effect of antioxidant supplements on the sustained virologic response has also been reported for chronic hepatitis C patients [138]. In addition, antioxidant treatment often involves a wide variety of drugs or mixtures and may have toxic or uncertain effects [133, 141]. Moreover, the pathology of viral diseases are ultimately the result of complex cellular reactions and host-virus interactions, and it remains to be clarified how antioxidant therapy modulates the cell redox state and treats HCV infection [141, 142]. Together, although many natural compounds have the ability to scavenge ROS or to activate endogenous defense systems, thus offering an indirect protection to the cell [143], with the current knowledge and uncertainties on the effect of antioxidants, targeting the redox-sensitive pathways as a complementary strategy to HCV therapy requires further investigations.

### 4.2. Oxidative Stress in DENV Infection

Similar to HCV, changes of redox status in the host cell are a common result of DENV infection. Currently, oxidative damage has been reported in severe DENV patients [144–146], indicating a correlation between oxidative stress and viral pathogenesis. DENV is shown to stimulate oxidative stress leading to chemokine RANTES secretion through the activating nuclear factor for IL-6 expression (NF-IL-6) signaling [85]. A recent study reports that changes in redox homeostasis and induction of oxidative stress are associated with the enhanced viral replication in monocytes from glucose-6-phosphate dehydrogenase- (G6PD-) deficient patients [14]. G6PD provides the reduced form of NADPH for various cellular reactions including GSH recycling and superoxide anion production via NADPH oxidase and nitric oxide (NO) synthesis; thus, G6PD deficiency weakens antioxidant defenses and increases oxidative stress in the cell. The G6PD deficiency alters the cellular redox into an abnormal state, and such oxidative state may benefit DENV replication. Thus, G6PD-deficient patients were found to have a higher virus load, and this may also be a link to the presence of severe dengue. This study laterally supports the link between cellular oxidative state and susceptibility to DENV infection. In primary human monocyte-derived dendritic cells (Mo-DC), DENV infection induces NOX-dependent oxidative stress that regulates the magnitude of the activation of innate antiviral immune responses and stimulates apoptosis [13]. More recently, the extent of oxidative stress has been associated with the disease severity in DENV-infected patients [146, 147]. The level of oxidative stress is found to be maximal in DSS followed by DHF, and its level is minimal in dengue fever [147], suggesting a correlation between the level of oxidative stress and DENV-induced pathogenesis.

In addition, DENV can manipulate antioxidant systems to favor its replication. In line with this notion, DENV serotype 2 (DENV2) infection alters the host intracellular GSH levels, and inhibition of GSH synthesis promotes DENV2 replication in liver cells and *in vivo* [148, 149], implicating antioxidant molecules as a potential therapeutic agent for the treatment of viral infections via the inhibition of oxidative stress. Induction of antioxidant enzyme HO-1 effectively inhibits DENV replication in Huh7 cells [150]. The inhibition of antioxidant pathways regulated by Nrf2 increases DENV-associated immune and apoptotic responses in Mo-DC [13]. Furthermore, the addition of garlic diallyl disulfide (DADS), diallyl sulfide (DAS), and alliin has been found to reduce DENV-mediated oxidative stress [151]. DAS is a selective inhibitor of cytochrome P450 2E1, which is shown to be upregulated by the HCV core and NS5A, respectively [119, 120]. These studies show that modulation of the antioxidant systems has a potential for the development of DENV therapy; however, more studies are required.

### 4.3. Oxidative Stress in the Infections of ZIKV

To date, there are very few reports about the effects of ROS on the ZIKV life cycle and ZIKV-induced pathogenesis; this is perhaps due to the fact that ZIKV began to attract research interest after its outbreak in recent years. A recent study shows that Nrf2-mediated signaling affects embryo survival, redox biology, and ZIKV susceptibility in the mosquito *Aedes aegypti* [152], thus linking oxidative stress and ZIKV infection. ZIKV infection can trigger ER stress (or UPR) in the cerebral cortex of infected postmortem human fetuses as well as in cultured human neural stem cells. Oxidative stress and ER stress are related states that occur frequently in diseases involving inflammation and viral infection [153]. One consequence of oxidative stress is the disruption of the correct oxidative environment within the ER, leading to the misfolding of proteins and ER stress [154, 155]. Meanwhile, protein misfolding in the ER may also result in excessive ROS production and therefore oxidative stress. The role of oxidative stress and the underlying molecular details during ZIKV infection needs to be further investigated.

### 4.4. Oxidative Stress in the Infections of JEV, WNV, and TBEV

Many continuous cell lines can support the production of JEV, and early studies show that JEV infection produces the toxic oxygen species in neutrophils [156], ROS intermediates in murine neuroblastoma cells [19], and superoxide anion and nitric oxide in rat cortical glial cells [157]. UV-inactivated JEV causes oxidative stress in mouse neuronal N18 cells [18]. Subsequently, in human astrocytoma and astroglioma cell lines, JEV infection is found to lead to ROS production and to regulate RANTES [158]. JEV induces massive inflammatory responses, which upregulates ROS [159]. The production of ROS is involved in the oxidative stress-induced apoptosis [160]. JEV infection of human promonocyte cells downregulates thioredoxin and induces ROS and ASK1-ERK/p38 MAPK signaling, and all are associated with JEV-induced apoptosis [20]. In the rat model, JEV is able to cause an imbalance of oxidants and antioxidant systems in different brain regions [161]. These studies suggest that oxidative stress contributes as a key factor in the pathogenesis of JEV infection. Besides, the therapeutic efficacy of antioxidants, including minocycline, arctigenin, fenofibrate, and curcumin, has been studied for their potential for the treatment of JEV infection [162].

Less studies are reported for the impact of WNV and TBEV on oxidative stress. The WNV infection of rabbit PBMCs induces the transcription of HO-1 and inducible NO synthase (iNOS), suggesting that oxidative stress may be involved [163]. However, natural WNV strain infections do not induce stress granules (SGs) and some WNV strains could inhibit the SG formation induced by arsenite treatment [22]. SGs are cytoplasmic messenger ribonucleoprotein structures (mRNPs) that are assembled in response to environmental stresses such as oxidative stress, and they contain an array of RNA-binding proteins, translation initiation factors, large and small ribosomal subunit protein components, and mRNAs. The mRNAs are primarily released from polysomes and sequestered into SGs as an adaptive response in eukaryotic cells [164]. The interaction of viral RNA and proteins with TIA-1/TIAR is found to interfere with the formation of SGs [165]. Thus, WNV induced both ROS and the antioxidant responses; however, the infected cells do not display characteristics of oxidative stress, since the antioxidant responses counteract the negative effects of ROS [22]. Thus, the cellular redox status is thought to be beneficial for the life cycle of WNV so far. The overexpression of TBEV NS1 triggers ROS production and activates the Nrf2/ARE pathway, yet its correlations with TBEV-induced damage of the central nervous system remains unclear [23].

## 5. Autophagy and Virus Infections

Viruses have evolved diverse strategies to exploit cellular processes and to escape from host defenses. One such central pathway is autophagy, an evolutionarily conserved mechanism that recycles damaged or unwanted cellular materials to maintain cell homeostasis. In resting cells, autophagy is suppressed by the mammalian target of rapamycin (mTOR) serine/threonine kinase. Autophagy is induced by cell stresses, such as starvation, ER stress, and pathogen infections, each of which leads to the dephosphorylation and inactivation of TOR [166]. Autophagy begins with the sequestration of intracellular components into a crescent-shaped isolation membrane [166, 167]. Isolation membranes contain autophagy proteins ATG5, ATG8 (known as LC3 in mammals), and ATG12. During the maturation process, the cytosolic microtubule-associated protein light chain 3 (LC3I) is conjugated to phosphatidylethanolamine, producing the membrane-bound, lipidated form of LC3II mediated by the ATG5-ATG12 conjugate [168]. This lipidated form of LC3 mediates membrane tethering and fusion to extend the isolation membrane by recruiting membranes from multiple sources, leading to the formation of large double-membrane vesicles known as autolysosomes [169], which subsequently fuse with endosomes/lysosomes where sequestered substrates are degraded [170, 171].

Since viral infections cause cell stress, autophagy is frequently induced as a byproduct of infections. However, virus-induced autophagy is not merely a passive process [170]. It has been shown that positive-stranded RNA viruses could manipulate autophagic machinery to evade host immune responses and facilitate viral replication [170, 172–175]. In accord with these phenomena, the increased formation of autophagosomes has been observed in HCV, DENV, and ZIKV-infected cells [176–178]. Additionally, autophagy can play dual roles of both proviral and antiviral functions depending on the virus type and the steps of the viral life cycle. Despite much efforts, the molecular mechanisms for how autophagy is induced during virus infection remain elusive. Many positive-stranded RNA viruses replicate in ER-mitochondria contact membranes, causing new membrane synthesis and rearrangement through the induction of UPR, many of the processes involving oxidative stress [179].

Although the role of autophagy in HCV and ZIKV infection is established (for recent reviews for HCV, see [180], and for ZIKV, see [181, 182]), the specific molecular mechanisms involved in the modulation of autophagy by *Flaviviridae* viruses have yet to be fully clarified. This review here focuses on the interplay between oxidative stress, autophagy, and virus-induced pathogenesis.

### 5.1. Oxidative Stress, Autophagy, and HCV Infection

Accumulating evidences have demonstrated that HCV infection induces autophagy in cell culture and in the hepatocytes of chronically infected patients [183]. Autophagy has been reported to function at the early stages of HCV infection such as the translation of incoming viral RNA. The induction of autophagy is required to sustain the survival of virally infected cells, which is an important characteristic of HCV chronic infection [184–187]. HCV RNA transfection blocks lysosomal fusion with autophagosomes, the membranes of which provide replication sites for HCV [188]. Several studies suggest that HCV induces the UPR *in vitro* [189–191] and in liver biopsies of HCV-infected patients [192]. The HCV-induced UPR in turn activates the autophagic pathway to promote viral RNA replication in human hepatoma cells [189]. The UPR-autophagy pathway could suppress the innate immune responses by repressing the production of interferon- (IFN-) *β* or of interferon-stimulated genes (ISGs) through the HCV-derived pathogen-associated molecular pattern (PAMP) [184, 189]. However, the molecular mechanism(s) for HCV-induced UPR remains unclear. Besides autophagy, HCV induces mitophagy to promote its persistent infection [193], and inhibition of mitophagy by knockdown of parkin attenuates HCV replication [81]. These results suggest that autophagy has mostly been attributed to a proviral function in HCV. However, a recent study demonstrates that an ER transmembrane protein SCOTIN recruited the NS5A protein to the autophagosomal compartment, where autophagic degradation of NS5A has taken place, thereby inhibiting HCV replication [194].

**Figure 1:**
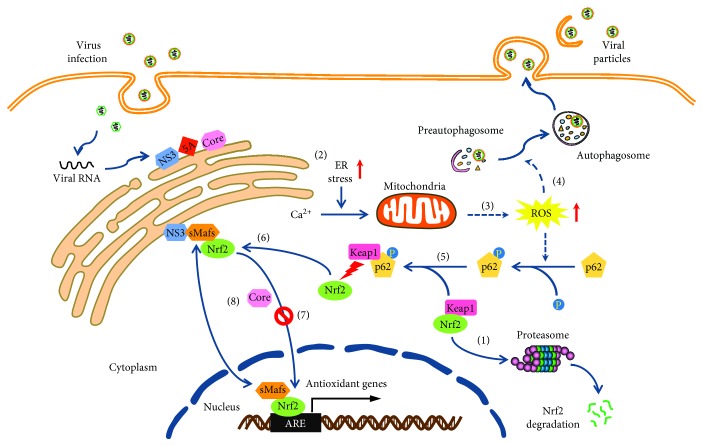
Proposed model for the interplay between HCV infection and ROS production, Nrf2 signaling, and autophagy. (1) Under homeostatic conditions, Keap1 sequesters Nrf2 in the cytosol, where it mediates proteasomal degradation of Nrf2. (2) HCV infection induces ER stress and alters ER calcium homeostasis. (3) The uptake of calcium in the mitochondria triggers ROS formation. (4) Oxidant stress induced by HCV infection induces the phosphorylation of p62 and autophagy. (5) Consequently, phosphorylated p62 increases its binding to Keap1, thereby releasing Nrf2 from the Keap1-Nrf2 complex. (6) Free Nrf2 is trapped via delocalized sMaf proteins that are associated with NS3 at the replication complex on the cytoplasmic face of the ER, (7) and thus preventing its translocation to the nucleus to induce antioxidant defenses, which in turn favors the release of viral particles. (8) In addition, HCV-induced sequestration of Nrf2 at the replication complex is core dependent, but how the core participates in this process remains unclear.

Oxidative stress is known to induce the autophagic machinery [195, 196]. As already mentioned, HCV infection is associated with elevated ROS levels and oxidative stress, which in turn activates autophagy to favor HCV particle release [197]. Oxidative stress induced by HCV infection triggers the phosphorylation of the autophagic adaptor protein p62 on Ser349 that is involved in the induction of autophagy. Consequently, phosphorylated p62 increases its binding to Keap1, thereby releasing Nrf2 from the Keap1-Nrf2 complex. In HCV-positive cells, sMafs are bound to NS3 at the replicon complexes on the cytoplasmic face of the ER, thus preventing its translocation to the nucleus. Free Nrf2 is trapped via delocalized sMaf proteins and is therefore prevented from its entry into the nucleus to induce antioxidant defenses, which in turn favors the release of viral particles. In addition, HCV-induced sequestration of Nrf2 at the replicon complex is core dependent, but how the core participates in this process remains to be investigated. Taken together, it is possible that the interplay between HCV-induced oxidative stress and the Nrf2 signaling elevates the ROS, leading to the induction of autophagy, thereby favoring HCV infection (Figure 1). Given that a functional association between a dysfunctional autophagy and Nrf2 pathway activation has been identified in HCC [198], oxidative stress by the inhibition of Nrf2 triggers autophagy and thus may be involved in chronic HCV infection-related HCC. It is worth noting that several papers also suggest that HCV can activate the Nrf2 pathway. On one hand, HCV-mediated Nrf2 activation contributes to the survival of HCV-infected Huh7 cells, which provides important clues to the understanding of the mechanisms of chronic liver disease induced by oxidative stress associated with HCV infection [199]. Conversely, at an early stage, the expression of HCV proteins in Huh7 cells induces a strong upregulation of the antioxidant defense system. These events may underlie the harmful effects of HCV-induced oxidative stress during the acute stage of hepatitis C [124]. Thus, the difference in Nrf2 status during HCV infection may depend on cell types and infection stages.

### 5.2. Oxidative Stress, Autophagy, and Flavivirus Infection

Since the initial characterization of autophagy in hepatocyte cells during DENV2 infection was reported in 2008 [200], several reports have demonstrated the proviral role of autophagy in DENV2 infection. NS1 is reported to partially colocalize with autophagosomes in hepatocytes, and inhibiting lysosomal fusion with autophagosomes increases viral replication, implying a role for immature autophagosomes in the DENV2 life cycle [201]. DENV3 also induces autophagy; however, the role of autophagy in its life cycle may differ from that for DENV2 as a lysosomal fusion inhibitor decreases DENV3 replication [202]. Moreover, a recent report shows that NS4A can induce autophagosome formation during DENV infection and protect the infected cells from apoptosis in renal epithelial cells and thus contribute to prolonged viral replication [203]. Additionally, DENV has been shown to induce the proliferation of LC3-containing membranes [204], and inhibition of cellular autophagy deranges DENV virion maturation [205]. Similar to HCV, the UPR-autophagy pathways have been shown to modulate the DENV-associated PAMP-induced innate immune response, implying that DENV exploits the UPR-autophagy pathways to escape from the innate immune response [189]. However, autophagy also has the potential to limit DENV replication. A recent study demonstrates that autophagy activity is increased and supports DENV replication during early infection, and at the later stage of infection specific autophagy suppression provides a viral replication advantage, suggesting the shifts of autophagy from a virus-supporting to a virus-suppressing process [176]. In addition to general autophagy, DENV-induced selective autophagy is termed as lipophagy, resulting in the release of free fatty acids (FFA), which undergoes oxidation in the mitochondria to generate ATP, and thus producing a metabolically favorable environment for viral replication [206]. Furthermore, DENV and ZIKV subvert reticulophagy through viral protease NS2B3-dependent cleavage of ER-localized reticulophagy receptor FAM134B [207].

To date, relatively few studies have been done on the role of autophagy under the infection of ZIKV. The precise molecular mechanisms involved in ZIKV-induced autophagy have not been fully elucidated. It has been demonstrated that autophagic vesicles are accumulated following ZIKV infection in both *in vitro* and *in vivo* models, suggesting the induction of autophagy in ZIKV infection. Similar to DENV, ZIKV can also induce the formation of LC3-containing membranes [175, 178, 208]. However, in contrast to DENV, where NS4A-induced autophagy confers protection from cell death, NS4A and NS4B of ZIKV dysregulate autophagy through AKT1-mTOR inhibition, leading to increased cell death and impeded neurogenesis in fetal neural stem cells [178]. Moreover, one study demonstrates that noncanonical secretory autophagy may contribute to the spread of ZIKV [209]. Recently, a study showed that ZIKV infection activates autophagic activity in human trophoblast cells and in the mouse placenta, and inhibition of the autophagy signaling reduces ZIKV vertical transmission and limits placental damage and fetal death, therefore providing a foundation for developing therapeutics targeting autophagy to reduce maternal-fetal transmission of ZIKV [210]. Furthermore, ZIKV triggers ER stress and UPR in the cerebral cortex of infected postmortem human fetuses as well as in cultured human neural stem cells, suggesting a potential role of oxidative stress in ZIKV-induced autophagy [211]. Given that oxidative stress plays a critical role in the induction of autophagy, in the future, more work is required to determine the interplay between oxidative stress and autophagy during DENV and ZIKV infections.

## 6. Oxidative Stress, DNA Damage, and Viral Infection

DNA is usually restricted within the nucleus and mitochondria of eukaryotic cells. RNA viruses are known to cause DNA damage, leading to the escape of self-DNA into the cytoplasm [212]. The presence of cytosolic DNA of mammalian cells serves as a danger signal that activates innate immune responses [213]. DNA damage can induce cell apoptosis, inflammatory immune responses, and deleterious mutations that increase the risk of tumorigenesis and contribute to the pathogenesis of RNA viruses [212, 214]. Therefore, the molecular basis for RNA virus-mediated DNA damage response remains an important area of study that will likely provide key insights into the modulation of host cell functions by these pathogens.

Growing evidences demonstrate that HCV increases the production of the hydroxyl radical and peroxynitrite close to the cell nucleus, therefore inflicting DNA damage. Further study shows that HCV increases the generation of the superoxide and H_2_O_2_ in proximity to the hepatocyte nucleus and that the source of ROS is primarily NOX1 and NOX4 [215]. During HCV infection, the core and NS3 proteins induce oxidative stress through the activation of iNOS, which in turn causes DNA damage and thereby results in the increased mutagenesis of cellular genes, including protooncogenes and tumor suppressor genes [216]. The core, E1, and NS3 proteins can also cause DNA damage and the activation of oncogene STAT-3 via ROS [126]. NS3/4A impairs DNA repair efficiency and enhances cellular sensitivity to DNA damage [217]. All of these contribute to the HCV-related hepatocarcinogenesis. Although the DNA repair mechanisms of the host cell have specifically evolved to counteract DNA damage, studies have suggested that the suppression of DNA repair might be a crucial consequence of chronic oxidative stress in HCV-infected cells [218]. The accumulation of DNA damage response in HCV-infected cells suggests that HCV-associated oxidative stress may overwhelm cellular antioxidant and DNA repair mechanisms, leading ultimately to neoplastic transformation. In line with this result, DNA damage is observed in PBMCs from HCV-infected patients and B cells *in vitro* [219]. The HCV core protein inhibits the formation of the complex of DNA damage sensor proteins and oxidatively damages DNA repair, resulting in DNA damage and hypersensitivity to DNA-damaging reagents. In addition, the HCV-triggered induction of ROS and the perturbation of DNA repair enzyme *NEIL1* expression may be involved in the progression of liver disease suggesting that antioxidant and antiviral therapies can reverse these deleterious effects of HCV in part by restoring the function of the DNA repair enzymes [220].

To date, only a few studies are reported on the role of DNA damage and the infection of DENV and ZIKV. A recent study demonstrates that DENV NS2B targets the DNA sensor cyclic GMP-AMP synthase (cGAS) for lysosomal degradation to avoid the detection of mtDNA during infection [221]. Similar to DENV, ZIKV infection induces a release of mtDNA which is identified as a ligand for cGAS, thereby eliciting inflammation to evade antiviral response [222]. As mtDNA is a major target of oxidative stress, the interplay between oxidative stress, mtDNA-associated inflammatory responses, and DENV or ZIKV-induced pathogenesis is a field of increasing interest.

## 7. Conclusions and Future Perspective

*Flaviviridae* virus infection induces oxidative stress, which affects both the cellular metabolism and the life cycle of the viruses. In the past decades, much progress has been made in understanding the interplay between oxidative stress and *Flaviviridae* viruses, especially for HCV and DENV. It is already known that oxidative stress plays dual roles in regulating viral replication; nevertheless, the underlying mechanisms are still unclear. More studies have shown that the Nrf2/ARE pathway is a common antioxidant system for HCV, DENV, and ZIKV; however, only few reports are done for JEV, TBEV, and WNV regarding oxidative stress and antioxidants. Arctigenin is found to have antioxidant and antiviral activities against the infection of JEV [223], and morpholino lowers the oxidative stress induced by JEV [224]. The TBEV NS1 protein both triggers ROS production and activates the Nrf2/ARE pathway [23]. Echinochrome A is reported to have antioxidant properties suppressing TBEV [225]. WNV could upregulate Nrf2 [22]. Fine-tuning the balance between ROS generation and detoxifying upon the infections of viruses requires more effort not only for JEV, TBEV, and WNV, which is hampered largely because of the lack of knowledge, but also for HCV, DENV, and ZIKV in the future. Knowledge gained from HCV, DENV, or other viruses may facilitate the study of the modulation of the oxidative stress in JEV, WNV, and TBEV infections. Once the mechanisms are elucidated, they may provide new insights into viral pathogenesis and the development of new therapeutics.

Understanding the role of oxidative stress and ROS-mediated DNA damage or autophagy might lead to new discoveries in pathology and novel strategies for interventions and clinical management. Moreover, the challenges involved in researching cellular ROS in viral infection are as follows: (1) Many positive-stranded RNA viruses replicate in ER-derived membranes, causing new membrane synthesis and rearrangement which are reorganized into viral replication organelles. While the architecture of these replication organelles is relatively defined, little is known about the viral and host factors orchestrating their biogenesis upon induction of oxidative stress. (2) HCV-induced ROS is required for the establishment of acute and chronic liver diseases. However, little is known about the role of oxidative stress in HCV-induced extrahepatic manifestations such as insulin resistance and neuronal disorders. (3) Although much of the available evidence supports the involvement of ROS in HCV and DENV-associated diseases, the interplay among viral proteins, cellular factors and enzymes for ROS production remains to be clarified. Thus, a better understanding of the underlying molecular mechanisms might help develop the study of the pathogenesis and antiviral therapies against the infections of *Flaviviridae* viruses. (4) In contrast to the chronic infection by HCV, apoptosis is usually the ultimate outcome of cells infected with DENV and ZIKV. It is of interest to determine why oxidative stress and associated molecular events such as autophagy and mtDNA damage can lead to different responses in the host cell. (5) Recent studies have shown that ZIKV has oncolytic activity against glioblastoma stem cells (GSCs), suggesting that the engineering of ZIKV may provide a therapeutic modality against glioblastoma [226–229]. As ZIKV selectively infects and kills GSCs relative to normal neuronal cells, it is of great interest to determine the role of oxidative stress in this process. (6) The role of oxidative stress in other flavivirus infections requires more effort.
